# Long-Term Outcomes after Pelvic Organ Prolapse Repair in Young Women

**DOI:** 10.3390/jcm11206112

**Published:** 2022-10-17

**Authors:** Marine Lallemant, Yasmine Clermont-Hama, Géraldine Giraudet, Chrystèle Rubod, Sophie Delplanque, Yohan Kerbage, Michel Cosson

**Affiliations:** Department of Gynecologic Surgery, Jeanne de Flandre University Hospital, 59000 Lille, France

**Keywords:** young women, POP repair, pelvic organ prolapse, recurrence, complications

## Abstract

The aim of the study was to describe the long-term outcomes of Pelvis Organ Prolapse (POP) repair in women under 40 years old. A retrospective chart review of all POP repairs performed in women ≤40 years old between January 1997 and December 2015 in the Gynecologic Surgery Department of Lille University Hospital was performed. Inclusion criteria were all women ≤40 years old who underwent a POP repair with a stage ≥2 POP according to the Baden and Walker classification. The study population was separated into three groups: a sacrohysteropexy group, a vaginal native tissue repair (NTR) group, and a transvaginal mesh surgery (VMS) group. The primary outcome was reoperation procedures for a symptomatic recurrent POP. Secondary outcomes were other complications. During the study period, 43 women ≤ 40 years old who underwent a POP repair were included and separated into three groups: 28 patients (68%), 8 patients (19%), and 7 patients (16%) in the sacrohysteropexy, VMS, and NTR groups respectively. The mean followup time was 83 ± 52 months. POP recurrence, reoperated or not, was essentially diagnosed in the VMS group (87.5%) and the NTR group (50%). POP recurrence repairs were performed for nine patients (21%): 7%, 62.5%, and 25% in the sacrohysteropexy, VMS, and NTR groups, respectively. Global reoperation concerned 10 patients (23%) whatever the type of POP surgery, mainly patients from the VMS group (75%) and from the NTR group (25%). It occurred in only 7% of patients from the sacrohysteropexy group. Two patients (4%) presented a vaginal exposure of the mesh (in the VMS group). De novo stress urinary incontinence was encountered by nine patients (21%): 29% and 12.5% in the sacrohysteropexy and NTR groups, respectively. Despite the risk of recurrence, POP repair should be proposed to young women in order to restore their quality of life. Vaginal native tissue repair or sacrohysteropexy should be performed after explaining to women the advantages and disadvantages of each procedure.

## 1. Introduction

Pelvic organ prolapse (POP) is a common disease in postmenopausal women. The incidence in the younger population is uncertain because many women suffering from POP do not seek a repair. According to Nygaard et al., the prevalence of POP in women under 40 years old is 1.6% [1]. Surgical repair of POP in young women is challenging because it must preserve the patient’s fertility and sexual functions while obtaining a satisfying and durable anatomic result. In the literature, the young age of women is often described as a risk factor for POP recurrence [2,3,4]. However, the long term results of POP repair in women under the age of 40 have rarely been studied [5,6]. In addition, there are no recommendations for the management of POP in young women. The aim of the study was to describe the long-term outcomes of POP repair in women under 40 years old.

## 2. Materials and Methods

We conducted a retrospective chart review of all POP repairs performed in women ≤40 years old between January 1997 and December 2015 in the Gynecologic Surgery Department of Lille University Hospital. The surgical techniques depended on the stage, the type of the POP, and the patient’s preference.

The inclusion criteria were all women ≤ 40 years old who underwent a POP repair with a stage ≥ 2 POP according to the Baden and Walker classification (Supplementary Table S1). POP surgeries were only performed if patients were symptomatic. Exclusion criteria were patients with a rectal prolapse and patients with missing data (no postoperative visit).

The study population was separated into three groups: a sacrohysteropexy group, a vaginal native tissue repair (NTR) group consisting mainly of posterior sacrospinofixations according to Richter or Richardson’s procedures, and a transvaginal mesh surgery (VMS) group. In this case, the mesh used was made of polypropylene (Prolift^®^, Pelvic Floor Repair System; Ethicon Women’s Health and Urology, Somerville, NJ, USA). Regardless the type of surgery, the surgery was performed under general anesthesia or loco-regional anesthesia. Postoperative care was standardized across surgeons. In the absence of any complication, the urinary catheter was removed, and the patient was allowed to ambulate the day after surgery, unless a laparotomy was performed.

Sacrohysteropexy was performed by laparoscopy or by laparotomy as described by Wattiez and Cosson [7,8]. Firstly, the pneumoperitoneum was established with a Veress needle. A 10-mm umbilical trocar was placed for the laparoscope. Two 5-mm right and left iliac trocars and one 10-mm suprapubic trocar were inserted. The surgeon was placed on the left side of the patient and the first assistant on the right. The peritoneum overlying the sacrum was opened and the anterior longitudinal sacral ligament was exposed. Anterior and/or posterior mesh was used. The vaginal fornices were dissected by mobilizing the bladder anteriorly and/or the rectum posteriorly, thanks to a curved metal vaginal manipulator. Anteriorly, dissection reached the bladder trigone, and posteriorly, the levator ani muscles were exposed. The mesh was sutured to the vaginal wall using absorbable sutures, with a digital control to avoid transfixing the vagina and to control the level of dissection. The anterior mesh was inserted through the right broad ligament before reaching the promontory. One or both tails of the mesh were suspended to the anterior sacral ligament by two permanent sutures. Meshes were peritonized to avoid the incarceration of a digestive loop.

The technique of posterior sacrospinofixation according to Richter began with a vaginal hysterectomy. Then, the posterior vaginal wall was exposed and infiltrated (mix of 30 mL Xylocaine 1% and 30 mL of isotonic saline). A posterior colpotomy was performed. The left or right pararectal fossa was dissected. The vagina was attached on one sacrospinous ligament. Then, the vagina was partially closed. The sacrospinous ligament fixation was tightened, and the vagina was completely closed. The Richardson procedure used the same technique but without the vaginal hysterectomy.

The transvaginal mesh repair technique was standardized, as described by Debodinance et al. [9]. An anterior and/or a posterior mesh was applied according to the prolapse type (anterior POP, posterior POP, or the complete floor) [10]. The anterior mesh was inserted between the bladder and the vagina and fixed laterally by four arms passing through the obturator foramen, near the tendinous arch of the pelvic fascia. The posterior mesh was placed between the rectum and the vagina with an arm passing through the ischiorectal fossa and fixed in the sacrospinous ligament. The mesh was made of non-absorbable monofilament polypropylene (Prolift Pelvic Floor Repair System; Ethicon Women’s Health and Urology, Somerville, NJ, USA).

Patients were identified from data collected by the Medical Information Systems Program. Information was extracted from paper and electronic hospital medical records. Demographic and medical data as well as histories were collected during the preoperative work-up. A physical examination was performed to determine the pelvic floor disorders. POP was classified according to the Baden and Walker classification [11]. Significant intraoperative complications (organ injuries or hemorrhage), early postoperative complications (hemorrhage, infection, or early reoperation before hospital discharge) or late postoperative complications (reoperation after hospital discharge and before postoperative visit) were recorded. All patients underwent a physical examination six weeks after surgery. Followup was performed once a year or more frequently, especially in the case of persistent or new symptoms related to the POP surgery, a failed surgery, or a recurrent prolapse. POP recurrence was defined as a Baden and Walker stage ≥ 2 in at least one vaginal compartment. Repeat surgeries for POP recurrence were performed based on functional and anatomical outcomes not on isolated anatomical outcomes.

The primary outcome was reoperation procedures for a symptomatic recurrent POP. Secondary outcomes were other complications: intraoperative organ trauma (bladder, bowel, and/or vagina), postoperative complications, such as vaginal hematoma and dyspareunia, postoperative POP recurrence (operated on or not), global reoperation and for mesh complications, and de novo stress urinary incontinence (SUI). Global reoperation included repeat surgeries for mesh complication or POP recurrence. Complications were classified according to the Clavien and Dindo classification [12].

According to French regulations, our study was exempted from a favorable opinion of an ethics committee, since this observational study used anonymized data from a medical database. In our center, patients were systematically informed that their medical data could be used for theoretical practice evaluation purposes and explicitly informed of the possibility of refusal.

Quantitative variables were expressed as mean ± standard deviation and qualitative variables as number of cases (percentage).

## 3. Results

**Figure 1:**
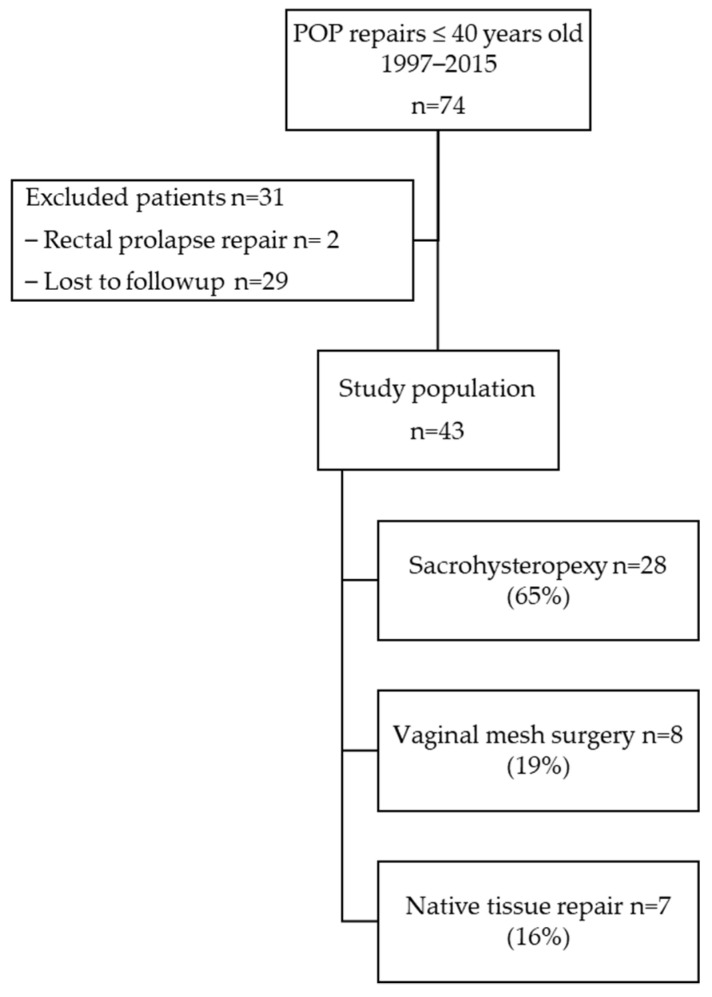
Flow chart.

**Figure 2:**
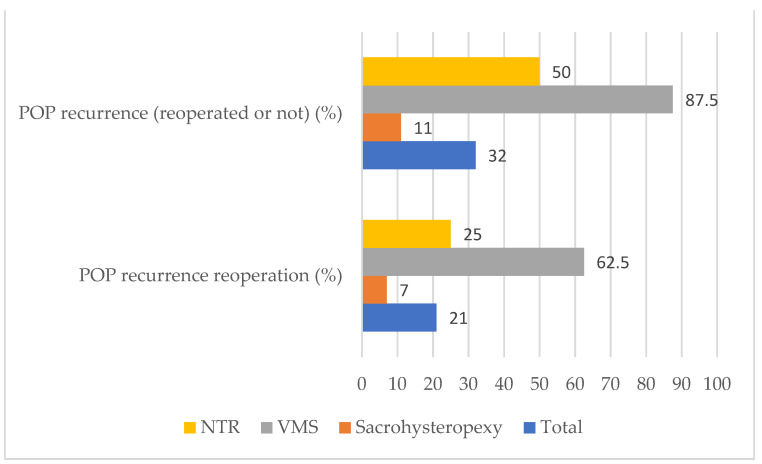
Long-term POP recurrence after a POP repair in women under 40 years old according to the surgical technique. POP: pelvic organ prolapse; VMS: vaginal mesh surgery; NTR: native tissue repair by vaginal route.

**Figure 3:**
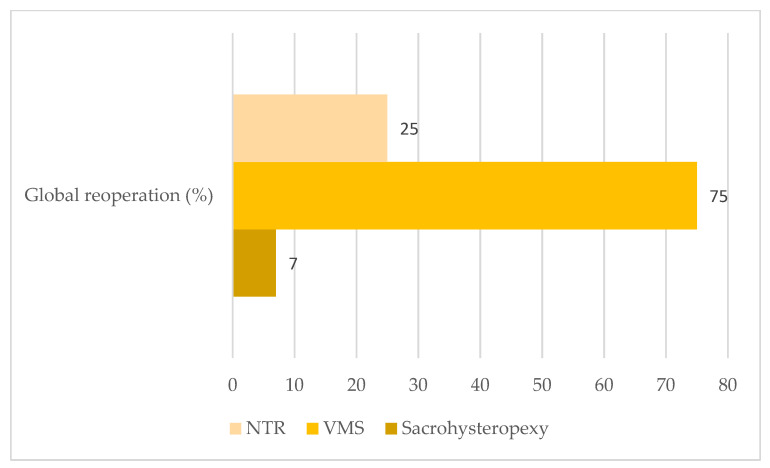
Global reoperation rates after a POP repair in women under 40 years old according to the surgical technique. VMS: vaginal mesh surgery; NTR: native tissue repair by vaginal route.

Between January 1997 and December 2015, 74 patients ≤40 years old underwent a POP repair. Among these, 31 patients were excluded: 2 patients suffered from a rectal prolapse, and 29 patients had missing data (lost to followup). Forty-three patients constituted the study population (Figure 1): 28 patients (68%), 8 patients (19%), and 7 patients (16%) in the sacrohysteropexy, VMS, and NTR groups respectively. Demographics, clinical examination, and surgery management characteristics are described in Table 1. The mean followup time was 83 ± 52 months. POP recurrence, reoperated or not, was essentially diagnosed in the VMS group (87.5%) and the NTR group (50%) (Table 2). POP recurrence repairs were performed for nine patients (21%): 7%, 62.5%, and 25% in the sacrohysteropexy, VMS, and NTR groups respectively (Figure 2). No intraoperative organ trauma was registered. Only one patient (in the VMS group) had a vaginal hematoma that was not reoperated. Postoperative dyspareunia was reported by only two patients (4%): one (4%) in the sacrohysteropexy group and one (12.5%) in the VMS group. No women reported pain in the ipsilateral gluteal region. Global reoperation concerned 10 patients (23%) whatever the type of POP surgery, mainly patients from the VMS group (75%) and from the NTR group (25%) (Figure 3). It occurred in only 7% of patients from the sacrohysteropexy group. Two patients (4%) presented a vaginal exposure of the mesh. All were from the VMS group (25%). Among these, only one underwent a surgical removal of the vaginal prosthetic mesh. De novo stress urinary incontinence was experienced by nine patients (21%): 29% (8/28) and 12.5% (1/8) in the sacrohysteropexy and NTR groups, respectively.

## 4. Discussion

In our study, women younger than 40 years old underwent the following surgeries in order of frequency: sacrohysteropexy (64%), vaginal mesh surgery (19%), and vaginal native tissue repair (16%). There are currently different surgical approaches for primary POP repair in young women. The first is that primary vaginal native tissue surgery could be preferred so that mesh repair could be used ulteriorly in case of recurrence. This approach is supported by Maher et al. who recommend native tissue repair as the first line of treatment [13]. The second is that given the possible weakness of the pelvic floor connective tissues and the lower risk of recurrence, sacrohysteropexy could be performed as a first-line procedure in young women. This concept is supported by Wagner et al. and Lucot et al. who demonstrated that sacrocolpopexy was associated with better long-term outcomes [14,15]. In our study, 19% of women underwent vaginal mesh surgery. Now, the use of synthetic vaginal mesh is no longer recommended since the FDA ordered manufacturers to stop distributing transvaginal surgical mesh in April 2019 [16]. Alternative surgeries such as pectopexy or lateral ligament suspension were not evaluated in this study because they were not performed in the first line in our center. According to Campagna et al.’s literature review, minimally invasive lateral suspension was safe (1% of grade ≥ 3 complications according to the Clavien–Dindo classification), efficient (90% of anatomic success in the apical compartment) and feasible [17]. However, there is a lack of well-designed, randomized, controlled trials assessing these techniques in the literature for POP repair, especially in young women.

In our study, POP recurrence occurred in 32% of patients and reoperation for POP recurrence in 21% of patients, regardless of the type of surgery. According to the literature, the rate of POP recurrence is about 30% in young women globally [2]. Friedman et al. reported a 36% recurrence rate in their meta-analysis [18]. In the study published by Hickman et al., the retreatment incidence was less than 10.3% [2]. However, their rate was lower than in the Lowenstein et al. study (26.9%) because of their shorter followup duration and loss to followup rate [3]. Other studies reported lower rates of recurrence but included a smaller and older study population and had a shorter followup period [19,20,21]. Some studies reported that young age was a risk factor for POP recurrence [22,23]. Vandendriessche et al. described a higher recurrence rate in young women who underwent a laparoscopic sacrohysteropexy [23]. Furthermore, some POP recurrences were probably not due to the surgical approach but rather due to the surgical strategy (i.e., anterior vaginal wall prolapse after isolated posterior vaginal mesh).

De novo stress urinary incontinence was reported by 21% of women, regardless of the type of surgery. It mainly occurred in women from the sacrohysteropexy group (29%). In the literature, it is estimated to range from 16 to 51% after surgical correction of prolapse [24,25,26,27].

POP recurrence (87.5%), reoperation for POP recurrence (62.5%), and global reoperation (75%) occurred often and more frequently in the vaginal mesh surgery group than in the sacrohysteropexy and native tissue repair groups. The samples size was too small for statistical comparison. However, even though meshes are no longer sold, these results confirmed that transvaginal mesh surgery should not be proposed as first-line POP therapy [28,29].

In our study, POP recurrence occurred in 11% of young women who underwent a sacrohysteropexy. However, secondary POP repair was performed in only 7% of the patients in this group. These results are consistent with those of Hickman et al. who studied POP recurrence in young women who had an abdominal surgery: overall incidence of recurrence 13.7% and overall retreatment incidence 5.9% [2].

In conclusion, morbidity was lower after sacrohysteropexy or vaginal native tissue repair. De novo SUI were more frequent in case of sacrohysteropexy. POP recurrence was slightly more reported in the NTR group. These results indicate that both procedures should be proposed to women explaining the advantages and disadvantages of each technique. In our center, we proposed a vaginal native tissue repair to women with a plan to become pregnant. Sacrohysteropexy is frequently chosen in case of POP recurrence or if the woman has no pregnancy plans.

In our study, two cases of postoperative dyspareunia after vaginal mesh surgery (one after an abdominal surgery and one after a transvaginal surgery) were reported. This could be due to a mesh retraction. In the literature, the rates of dyspareunia were higher than 4% [30]. In Hickman et al.’s study, 31.2% of women reported pain with intercourse [2].

Our study is original in describing the long-term outcomes of POP repair in women ≤40 years old. The major limitation of this study was its retrospective design and lack of randomization. The retrospective collection of baseline data generally misestimates adverse events and introduces measurement bias. In addition, statistical comparison of the three surgical techniques was not possible because of the small sample of patients included in the followup analysis. Pregnancy and childbirth after primary POP repair were not specified. This could be a confusion bias. Finally, our study assumed that the surgical technique was stable over the study period. However, even though the surgeries were performed in a single reference center over a period of 18 years by an experienced team, guaranteeing a relative homogeneity of procedures, slight changes in the surgical technique necessarily occurred progressively.

## 5. Conclusions

Despite the risk of recurrence, POP repair should be proposed to young women in order to restore their quality of life. Vaginal native tissue repair or sacrohysteropexy can be performed after explaining to women the advantages and disadvantages of each procedure.

## Figures and Tables

**Table 1:** Population characteristics.

Population Characteristics	*n* = 43
Age (years)	35 ± 4 (28–40)
BMI (kg/m^2^)	25.3 ± 5.3 (17.2–42)
Parity	2 (0–6)
Length of followup (months)	83 ± 52 (22–226)
POP stage (Baden and Walker classification) in at least one compartment:	
Stage 2	12 (28)
Stage ≥ 3	31 (72)
Type of POP repair	
Sacrohysteropexy	28 (65)
Anterior and posterior meshes	27 (96)
Posterior mesh only	1 (4)
Laparoscopic surgery	27 (96)
Laparotomy surgery	1 (4)
Vaginal mesh surgery	8 (19)
Anterior and posterior meshes	3 (37.5)
Posterior mesh only	5 (62.5)
Native tissue repair	8 (19)
Posterior sacrospinofixation according to Richter	1 (14)
Richardson’s procedure	5 (71)
Posterior colporrhaphy and perineorrhaphy	1 (14)
Concomitant total hysterectomy	16 (37)
Associated SUI surgery	11 (26)

Quantitative variables are expressed as mean ± standard deviation (range) and qualitative variables as number of cases (percentage). BMI: body mass index; POP: pelvic organ prolapse; SUI: stress urinary surgery.

**Table 2:** Long-term complications according to the Clavien–Dindo classification after a POP repair in women under 40 years old.

	Total *n* = 43	Sacrohysteropexy *n* = 28	VMS *n* = 8	NTR *n* = 8
Intraoperative complications	0			
Grade I and II				
Vaginal hematoma	1 (2)	0	1 (12.5)	0
Dyspareunia	2 (4)	1 (4%)	1 (12.5)	0
Non-reoperated POP recurrence	5 (12)	1 (4%)	2 (25)	2 (25)
Mesh exposure	1 (2)		1 (12.5)	
Grade III				
Global reoperation	10 (23)	2 (7)	6 (75)	2 (25)
POP recurrence reoperation	9 (21)	2 (7)	5 (62.5)	2 (25)
Mesh related reoperation	1 (2)	0	1 (12.5)	0
Grade IV or V	0			

Qualitative variables are expressed as number of cases (percentage). POP: pelvic organ prolapse; VMS: vaginal mesh surgery; NTR: native tissue repair by vaginal route.

## Data Availability

The data used to support the findings of this study are available from the corresponding author upon request.

## Supplementary Materials

The following supporting information can be downloaded at: https://www.mdpi.com/article/10.3390/jcm11206112/s1, Supplementary Table S1. Baden and Walker prolapse grading [11].
