# Supplementary material for: Long-Term Outcomes after Pelvic Organ Prolapse Repair in Young Women
Source: J Clin Med. 2022 Oct 17;11(20):6112. doi: 10.3390/jcm11206112 (PMC9605202; doi:10.3390/jcm11206112)
Supplement: Supplementary file 1 [file jcm-11-06112-s001.zip › jcm-1962082-supplementary.pdf]

Supplementary Table S1. Baden and Walker prolapse grading [11]

Stage	Position of structure
0	Normal
1	Halfway to hymen
2	At hymen
3	Halfway past hymen
4	Maximum descent
